# Effect of selective-precipitations process on the corrosion resistance and hardness of dual-phase high-carbon steel

**DOI:** 10.1038/s41598-019-52228-z

**Published:** 2019-10-30

**Authors:** Wilson Handoko, Aayush Anurag, Farshid Pahlevani, Rumana Hossain, Karen Privat, Veena Sahajwalla

**Affiliations:** 1 Centre for Sustainable Materials Research and Technology (SMaRT Centre), School of Materials Science and Engineering, University of New South Wales (UNSW Sydney), Sydney, NSW 2052 Australia; 2 Exchange student from Department of Metallurgical Engineering and Materials Science at Indian Institute of Technology, Bombay, India; 3 Electron Microscope Unit, Mark Wainwright Analytical Centre, University of New South Wales (UNSW Sydney), Sydney, NSW 2052 Australia

**Keywords:** Metals and alloys, Microscopy

## Abstract

It is commonly known that precipitation of secondary phase in non-ferrous alloys will affect the mechanical properties of them. But due to the nature of dual-phase low-alloy high-carbon steel and its high potential of precipitation of cementite, there is limited study on tailoring the mechanical and corrosion properties of this grade of steel by controlling the precipitation of different phases. Predicting and controlling precipitation behaviour on this grade of steel is of great importance towards producing more advanced applications using this low-cost alloy. In this study the new concept of selective-precipitation process for controlling the mechanical and corrosion behaviour of dual-phase low-alloy high-carbon steel has been introduced. We have investigated the precipitation of different phases using *in-situ* observation ultra-high temperature confocal scanning laser microscopy, image analyser – ImageJ, scanning electron microscopy with energy dispersive spectroscopy (SEM/EDS) and electron probe microanalysis (EPMA). Volume fraction of each phase including retained austenite, martensite and precipitated phases was determined by X-ray diffraction (XRD), electrochemical corrosion test by Tafel extrapolation method and hardness performance by nanoindentation hardness measurement. The experimental results demonstrated that, by controlling the precipitations inside the matrix and at grain boundaries through heat treatment, we can increase the hardness of steel from 7.81 GPa to 11.4 GPa. Also, corrosion resistance of steel at different condition has been investigated. This new approach will open new possibility of using this low-cost steel for high performance applications.

## Introduction

Tailoring the hardness and corrosion resistance of dual-phase low-alloy high-carbon steel is gathering huge attention recently because of low-cost production of this grade of steel and its exceptional properties^1,2^. Utilisation of this high strength, hardness and wear resistance steels in engineering products reduces metal intensity, promoting cost-effectiveness due to its low energy consumption^3^, thus reducing the issues of serious environmental pollution and resource scarcity^4–7^. The superior properties can be achieved either by adding alloying elements with various complex manufacturing processes, which will burden the production cost^8^; or can be obtained by modifying microstructures through heat treatment without changing the chemical composition^9^ which is seen to be more effective method.

In this study, we have used dual-phase high-carbon steels which consist of retained austenitic and martensitic phases. By understanding the effect of different heat treatment conditions, especially its austenitising time and temperature and their effect on corrosion resistance and hardness, gives us the ability to tailor these properties during production without changing the existing composition of steel. During steel making process, because of high carbon content in this grade of steel, the presence of carbides and inclusions are substantially inevitable. During the deformation stage of this grade of steel, by selecting appropriate austenitisation temperature, we will be able to reduce the number of carbide phases present in the structure. On the other hand, increasing austenitising temperature and isothermal holding time will increase the production cost and at the same time will increase the grain size and formation of sulphide-based phases^10,11^. Population and size of secondary phase precipitations can strikingly influence the corrosion resistance and hardness properties of high-carbon steels.

In addition, it is very likely for MnS precipitations to be generated during solidification process of steel, due to very low solubility between S and Fe elements at room temperature^12^. Other inclusions formations such as MnC formation in the steel is considerably unavoidable as the C content in high-carbon steel is relatively high, this can cause Mn and C to form manganese carbide (MnC) that mainly corresponds to different temperature, activity and diffusivity of C. Other carbides such as Fe_3_C occurred when C element is depleted from its solid solution in α-Fe matrix; sometimes it forms a belt like carbide chain on and along the grain boundaries^13^. Additionally, the amount, average size and volume percentage of secondary phase precipitated were attributed to heating temperature, dwelling time and quenching rate, in which dispersed inclusions in steels remained after solidification process^14^. Presence of these precipitations in steel escalated the propensity towards pit formation that associated perturbations on an atomic scale^15,16^.

In the current work we have investigated the influence of selective-precipitation process under different heat treatment condition. By changing the population and size of formed MnS, Fe_3_C and MnC inclusions through different heat treatment approach, high-carbon low-alloy steel with the same composition but different hardness and corrosion properties has been produced. In this study we carefully characterised the effect of grain size, precipitation type and volume fraction of each phase including MnS, Fe_3_C and MnC on its corrosion behaviour into 3.5 wt% NaCl solution and hardness test of high-carbon steel samples. *In-situ* observation by ultra-high temperature confocal scanning laser microscopy had been implemented to analyse its surface evolution that occurred on the formation of precipitations during different austenitising temperature. Morphological and chemical analysis were conducted by scanning electron microscopy with energy dispersive spectroscopy (SEM/EDS) and electron probe microanalysis (EPMA). Each phase volume percentage – retained austenite, martensite, MnS and MnC inclusions, was resolved by X-ray diffraction (XRD), electrochemical measurements by Tafel extrapolation method and hardness properties by nanoindentation hardness test.

## Experimental Procedures

### Materials preparation

High-carbon steel samples with chemical composition of 1.0 C, 1.0Mn, 0.65Cr, 0.25Si, 0.2Cu, (in wt%) has been utilised in this research. All samples were cut to two pieces with 12 × 12 × 4 mm dimension by Struers Accutom-50, feeding speed of 7 mm/min. Samples were then grinded and polished by SiC paper up to 4,000 fine grits – Struers TegraPol-21 and by diamond suspension up to 1 μm – Struers TegraPol-15 respectively to fit 10 mm-diameter Al_2_O_3_ crucible for ultra-high temperature confocal scanning laser microscopy experiments. Mirror-polished steel samples were heated, and *in-situ* observed through confocal microscopy – CSLM VL2000DX from Lasertec Corporation (Yokohama, Japan) with IR furnace SVF17-SP from Yonekura Corporation (Kanagawa, Japan). A sample was placed at the top focal point inside gold-coated elliptic chamber and halogen lamp, which acted as heat source producing infrared rays, which was located at the bottom focal point. A thermocouple was placed below the sample for temperature control. A laser beam with 408 nm wavelength was used for capturing the confocal images of the steel at a frame rate of 30fps along with 1024 × 1024 pixel resolution. Before performing the experiment, chamber was perfomed O_2_ and H_2_O free to prevent oxidation of sample at high temperature. Chamber was first evacuated using an oil-sealed rotary pump for around 3 mins and then filled with high-purity Ar gas for around 3 mins. The pressure of Ar gas was kept around 7 kPa higher than atmospheric pressure for the entire process. This process was repeated four times with highly pure argon gas passing through clean column and purifier being used, which reduced O_2_ and H_2_O content to less than 1.0 ppm. HiTOS-D software was used to setting up the heating parameters (900 °C and 1200 °C with dwelling time of 120 mins, quenching rate of −3000 °C/min) and real time monitoring of the temperatures.

### Analytical methods

Formation of secondary phase precipitation at heating temperature of 900 °C and 1200 °C (labelled as sample-900 °C and sample-1200 °C) was classified and its volume fraction was defined through image analyser, ImageJ. Scanning Electron Microscopy with Energy Dispersive Spectroscopy (SEM/EDS), Hitachi S3400I was performed to analyse surface morphology and composition of precipitations by mapping mode. Chemical analyses on steel surface by Electron Probe Microanalysis (EPMA) – JEOL JXA-8500F was used to characterise various elements in detail that created the precipitation and its surrounding. X-ray Diffraction (XRD) analysis was implemented to accurately define the volume percentage of each phase (retained austenite, martensite and secondary phase precipitations) on heat-treated steel samples and data were compiled and processed by HighScore Plus Malvern Panalytical software. Electrochemical test experiments were implemented through Versatile Multipotentiostat VSP300 and collective quantitative data was compiled by EC-Lab v11.10. The standard different connectors (saturated calomel electrode (SCE) for reference electrode, platinum electrode for counter electrode and high-carbon steel sample for working electrode) were employed and connected to this instrument. 3.5 wt. % NaCl solution – deaerated by purging with N_2_ gas to remove dissolve gases, was utilised in the electrochemical measurements and temperature was kept constant at 24 ± 1 °C. Tested region of steel specimen was 1 cm^2^ area in open circuit potential (OCP) condition and immersion time for 2 h, with potential ranges between −0.25 V and 0.25 V, scan rate 4 × 10^−4^ V/s and bandwidth of 10. Average values of corrosion potential, E_corr_ and its current density, i_corr_ were defined from collected Tafel quantitative data. Mechanical properties of sample i.e. elastic modulus (E) and nano hardness (H), were obtained from nanoindentation tests carried out using a Hysitron Triboindenter (Hysitron TI900, Minneapolis, USA) with a Berkovich diamond indenter (nominal angle of 65.3° and radius of 200 nm). This instrument was first calibrated by performing indentations on standard fused silica and a standard aluminium sample. The indentation load used during testing was increased linearly up to 8 mN, held at 8 mN for 5 s, and then it was decreased to zero.

## Results and Discussions

### Ultra-High Temperature Confocal Scanning Laser Microscopy with Grain Size Calculation

**Figure 1:**
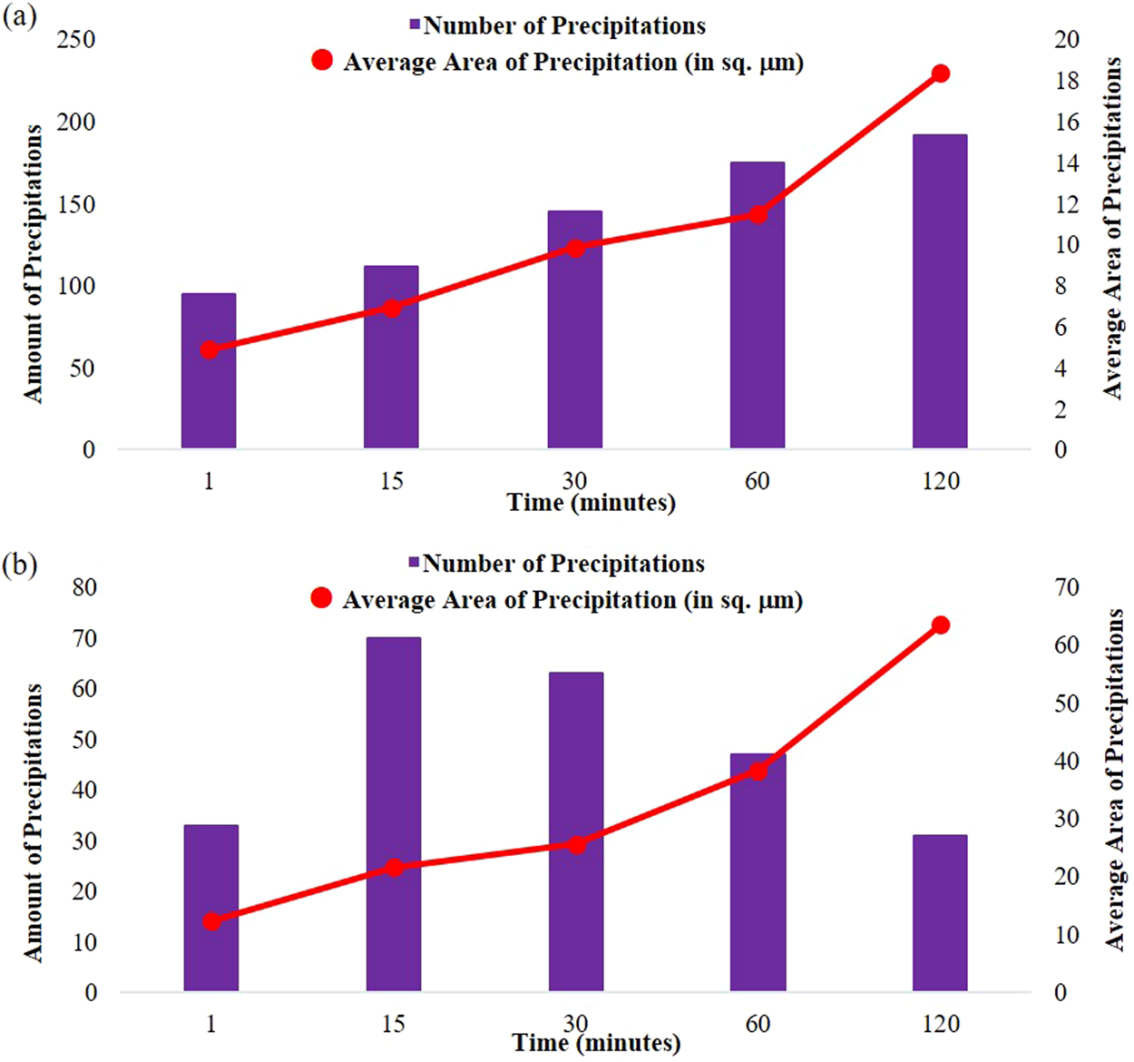
Comparison on amount and average area of precipitations of high-carbon steel samples at heat treatment for (**a**) sample-900 °C and (**b**) sample-1200 °C.

For the *in-situ* observation at different austenitisation temperature of dual-phase high-carbon steel samples, ultra-high temperature confocal scanning laser microscope was employed for heat treatment experiments. It was utilised to generate a sequence of images at different intervals of time for each sample and those recorded images represented corresponding changes that were then analysed using image analyser to generate quantitative data about the change in grain size and formation of secondary phase precipitations over designated time. During heat treatment process, there were formation of precipitations that had appeared in the matrix, on and along the grain boundaries on both steel samples. Figure 1 summarises the generated amount and average area of precipitations (by image analyser, ImageJ software) at temperature of 900 °C and 1200 °C. In a 1,048,576 µm^2^ recorded area of one image on steel surface, data was analysed and calculated.

**Figure 2:**
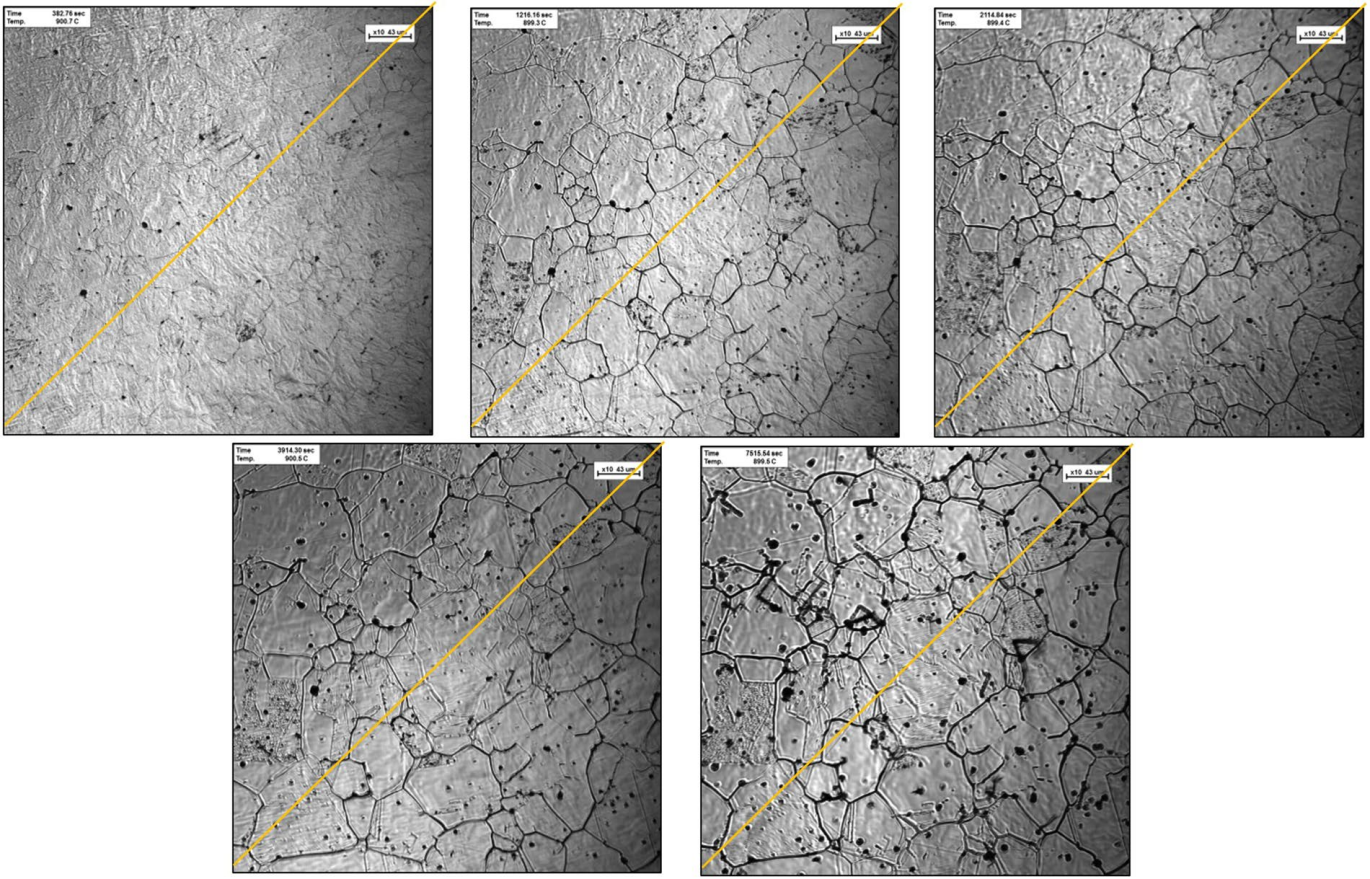
Ultra-high temperature confocal scanning laser microscopy images of heat-treated high-carbon steel samples at temperature of 900 °C with different interval (**a**) 1 min, (**b**) 15 mins, (**c**) 30 mins, (**d**) 60 mins and (**e**) 120 mins. Yellow line overlayed on each image was presented to determine grain size, using intercept method.

On sample-900 °C, there was initially less amount of “dark spots” or secondary phase precipitations, as presented in Fig. 2. The yellow line overlayed on each image was presented for grain size measurement as per agreement with ASTM standard, intercept method^17^. As the heating time increased, a significant amount of small inclusions appeared on the surface of the steel. It can be observed that while the average area per precipitate increased over time, the total number of precipitates decreased, indicating that while some of the inclusions diffused into the matrix, others agglomerated to form larger size of inclusions.

**Figure 3:**
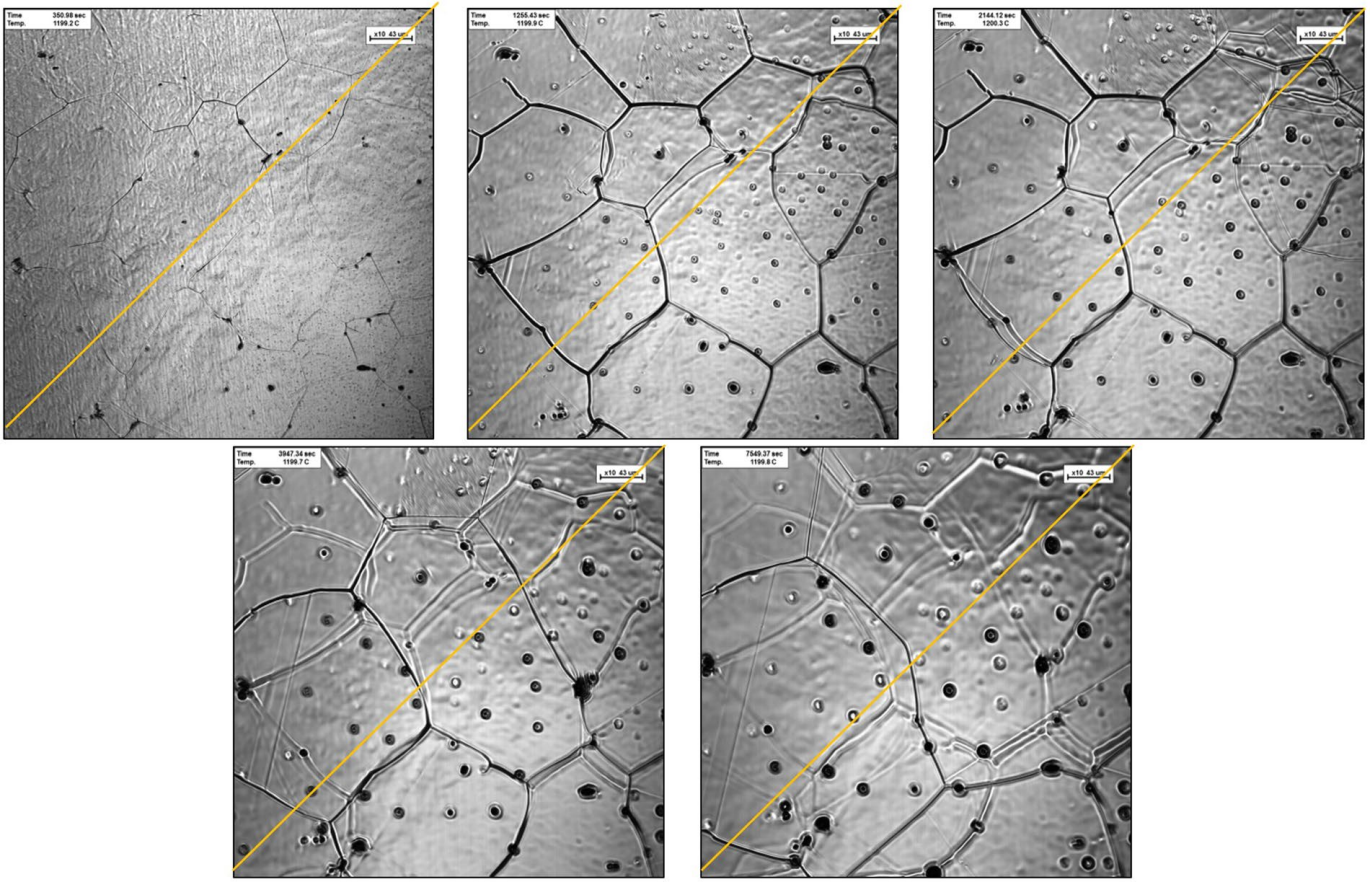
Ultra-high temperature confocal scanning laser microscopy images of heat-treated high-carbon steel samples at temperature of 1200 °C with different interval (**a**) 1 min, (**b**) 15 mins, (**c**) 30 mins, (**d**) 60 mins and (**e**) 120 mins. Yellow line overlayed on each image was presented to determine grain size, using intercept method.

With time, this occurred due to the dissolution of the inclusion in the matrix as higher diffusivity of inclusions at higher temperature. Moreover, some secondary phase precipitation dissolved in the matrix over time, whereas at the same time some others nucleated and underwent growth phase. A similar trend was occurred on sample-1200 °C on Fig. 3, with larger grain size and shorter grain boundary during the heat treatment. As the heating temperature increased, it will influence the grain growth correspond such that the average size of the grain boundary decreased yet the average area and diffused precipitation increased over a period^18,19^.

A nine images were collected and average grain size value of each steel sample against ASTM grain size numbers were calculated using standard formula, where *G* refers to the grain size value, *L* defines as mean intercept grain size in mm unit^17^, as follows:

$$G=-\,3.2877-6.6439lo{g}_{10}(\frac{L}{1000})$$

**Figure 4:**
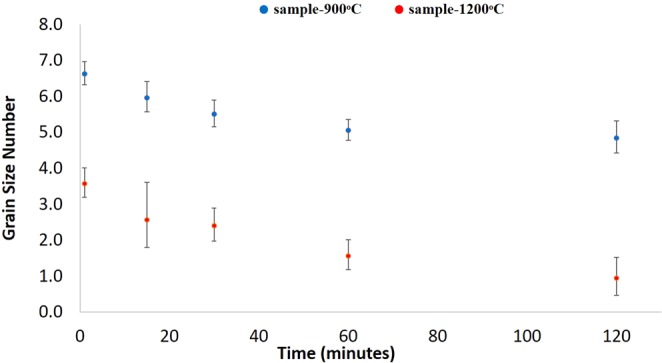
Comparison of the effect on heat treatment temperature at 900 °C and 1200 °C on ASTM grain size value of high-carbon steel as a function of time.

From the Fig. 4. It was predicted that as the heating temperature increase, the ASTM grain size decreased, meaning that the average grain size increased. Conversion of ASTM grain size to mean intercept grain size on sample-900 °C and sample-1200 °C equated to approximately 40.1 µm and 119 µm, as the smallest with time. On the other hand, the largest grain size on sample-900 °C and sample-1200 °C measured to around 86.2 µm and 254 µm.

### SEM/EDS analysis

**Figure 5:**
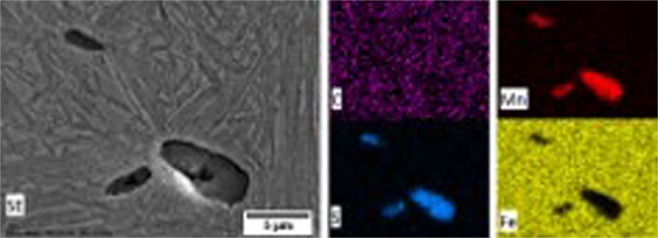
SEM image of precipitations with EDS analysis on C, Mn, S and Fe elements, showing MnS inclusions on high-carbon steel after heat treatment at 900 °C.

**Figure 6:**
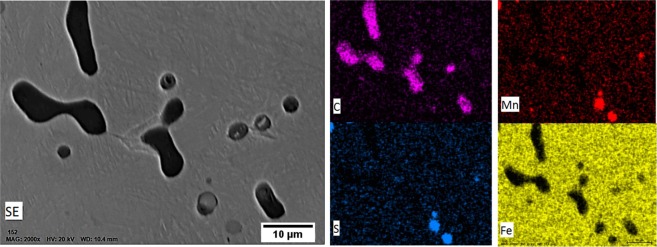
SEM image of precipitations with EDS analysis on C, Mn, S and Fe elements, showing MnS and MnC inclusions on high-carbon steel after heat treatment at 1200 °C.

After findings of existence on precipitation at different heat treatment temperature, the micro-analysis approach is necessary to analyse its final morphology and chemical analysis of these precipitations. To fully understand the effects of these selective-precipitations on corrosion behaviour and hardness performance of high-carbon steel, it is imperative to characterise these precipitations at different austenitisation temperature by using SEM/EDS analysis. On sample-900 °C, most of the precipitations were analysed and found to be high concentrated in Mn and S elements, providing strong evidence of the presence of manganese sulphide (MnS) inclusion, as shown in Fig. 5. The Mn-depleted zone and MnS precipitaions can be formed, corresponding to quenching rate, isothermal holding time and S capacity of the precipitation^20^. In addition, it was found that few micron-sized of MnC inclusions in very low concentration occurred in few areas of the steel surface and traces of other elements such as Al, Si, Cr, Ca and Ti were not detected on the surface of sample. Previous studies had shown that depending on the heat treatment temperature, dwelling time and cooling rate, nanometre-sized MnC inclusions can be precipitated^21,22^. Whereas on sample-1200 °C, it can be inferred that high-concentrated C, Mn, S and Fe elements were detected in this area, indicating the formation of MnS and MnC precipitations on this grade of high-carbon steel sample as shown in Fig. 6. Further analysis in detail is essential to observe different type of secondary phase precipitations, i.e. grain boundary form through Electron Probe Microanalysis (EPMA).

### EPMA analysis

**Figure 7:**
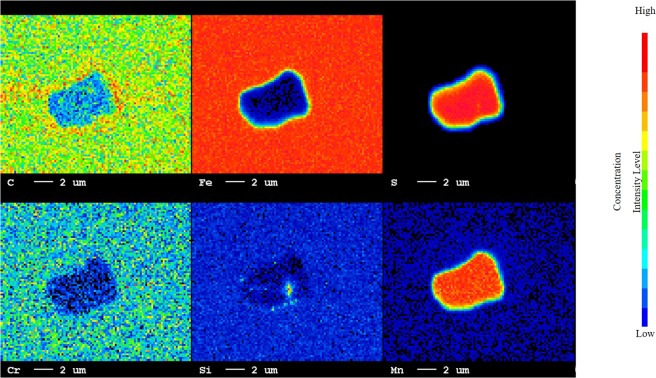
EPMA micro-analysis on MnS inclusion occurred on heat-treated high-carbon steel sample at 900 °C.

**Figure 8:**
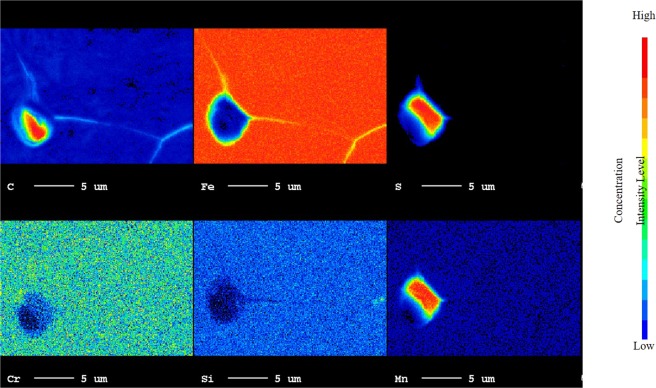
EPMA micro-analysis on MnS and MnC inclusions with Fe_3_C on and along the grain boundary on heat-treated high-carbon steel sample at temperature of 1200 °C.

A non-destructive chemical analysis of steel was conducted by EPMA analysis to determine different phases, precipitations and grain boundary in a matrix. In Fig. 7, it can be observed that precipitation of MnS phase surrounded by Fe_3_C phase occurred on sample-900 °C. Presence of low quantity of Si on surface of steel was believed to be trapped SiC from polishing paper. This is very interesting behaviour as it shows the gradual change from sulphide precipitation to carbide. In sample-1200 °C, a selective precipitation of two phase has happened which is MnS and MnC and grain boundaries carbides. Existence of high-level of Mn, S, Fe and C concentration was spotted along the grain boundary on sample-1200 °C, (Fe_3_C) and inclusions (MnS and MnC) on the surface of this steel, as presented in Fig. 8. Grain boundaries carbides can cause an increase in corrosion rate, since intergranular attack (IGA), pit growth and grain boundary corrosion will initiate from these inclusions^23,24^. However, these inclusions can have positive affect on hardness properties as micrometre-sized carbides hardened the steels^25,26^. Further investigation is required to determine its volume percentage of MnS and MnC inclusion through XRD analysis. This finding of different inclusion and its volume percentage will have substantial influence on the corrosion resistance and hardness properties of high-carbon steels.

### XRD analysis

**Figure 9:**
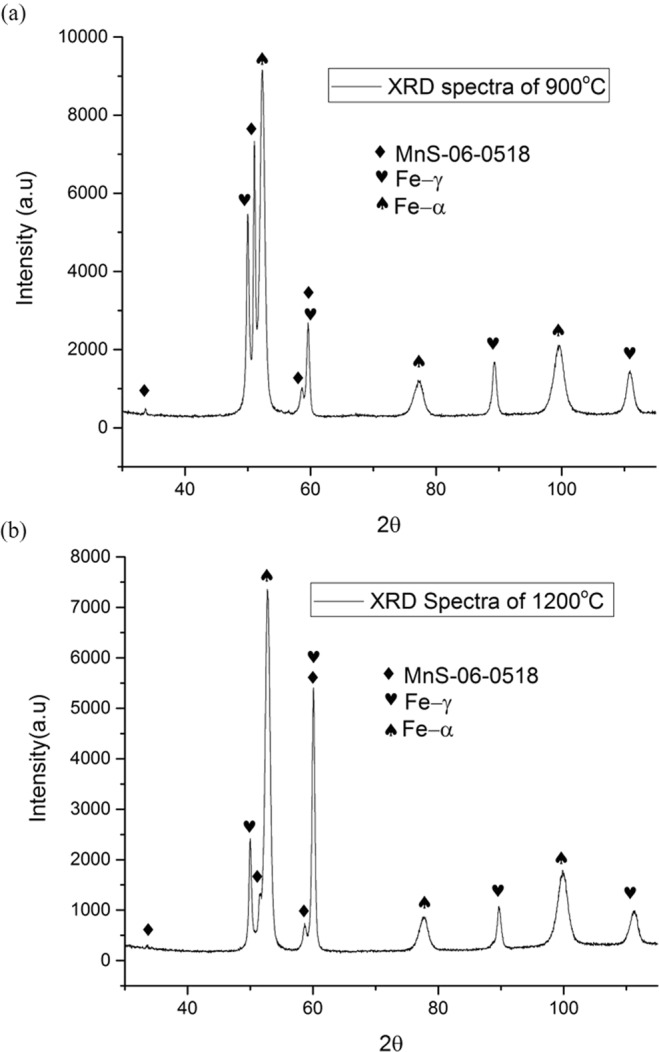
Comparison of XRD spectra to determine different phases on heat-treated high-carbon steel samples at temperature of (**a**) 900 °C and (**b**) 1200 °C.

XRD analysis was conducted to precisely determine volume percentage of each phase - martensite, retained austenite and precipitations through its unique crystallinity patterns, as shown in Fig. 9(a,b).

It was performed on both final samples of sample-900 °C and sample-1200 °C obtained after the austenitisation heat treatment process. A comparison volume percentage on each phase at different heating temperature is exhibited in Table 1. On sample-900 °C, retained austenite phase possessed higher volume percentage of about 1.4% higher than sample-1200 °C, while its martensite phase accounted less volume percentage around 4.8% than heat-treated steel sample at temperature of 1200 °C. This phenomenon was consistent as per agreement with Fe-C phase diagram system at different austenitisating temperature and constant quenching rate^27,28^. The percentage of the precipitation that was formed in sample-900 °C constituted of the MnS in which was approximately 7%. On the other hand, on sample-1200 °C, MnS inclusion existed with volume percentage of around 3.6%. From this XRD analysis, different volume percentage on each phase can have different influence on its corrosion resistance and hardness properties. Electrochemical corrosion test and nanoindentation hardness test methods were carried out to assess the effect of secondary phase precipitations on different heat-treated temperature on high-carbon steels properties in detail.

**Table 1:** Volume percentage of different phase extracted from XRD patterns on heat-treated high-carbon steel samples at temperature of 900 °C and 1200 °C.

Phase-ID\Temperature	900 °C	1200 °C
Retained Austenite	12.8	11.4
Martensite	80.2	85.0
Manganese Sulphide (MnS)	7.0	3.6

### Electrochemical test

**Figure 10:**
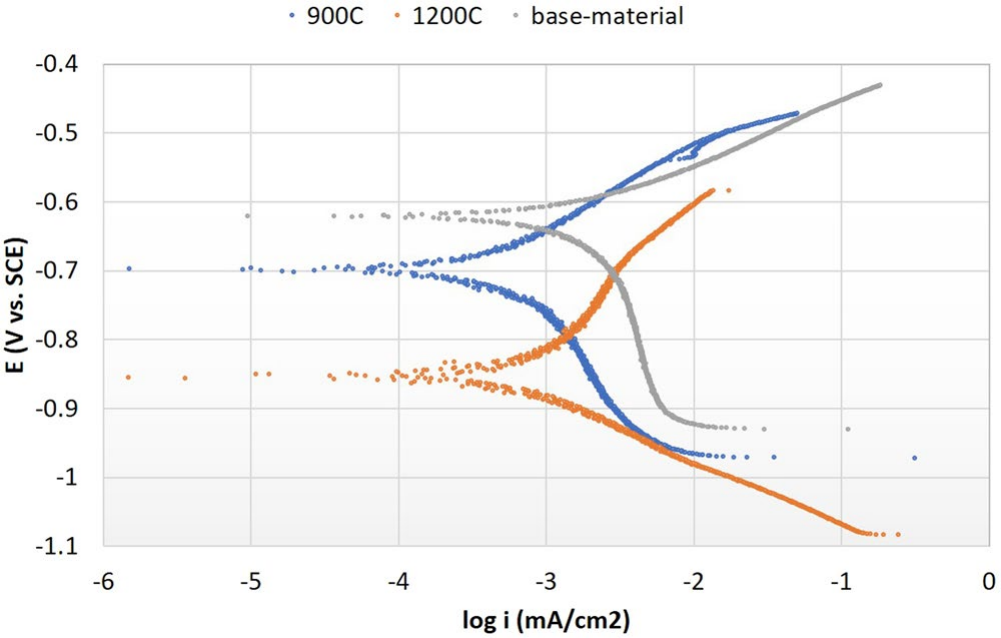
Tafel polarisation curves on the effect of secondary phase precipitation through different heat treatment temperature compared to base-material high-carbon steel.

Acquired quantitative data from electrochemical test method was compiled to generate average Tafel polarisation plots. Each i_corr_ value can be determined through the cross-path between the slopes of anodic (top branch - discharging) as metal oxidation or dissolution reaction and cathodic (bottom branch - charging) as H_2_ transformation process, horizontally perpendicular to E_corr_ in Tafel plot. From Fig. 10, original steel (before heat-treated) or “base-material” presented better corrosion resistance properties than both 900 °C and 1200 °C, as it possessed lower i_corr_ value and more positive E_corr_ value. Summary of the electrochemical test quantitative data is shown in Table 2. Polarisation behaviour, i_corr_ value for base-material accounted as 8.38% and 9.84% lower than sample-900 °C and sample-1200 °C accordingly. For E_corr_ value, base-material contributed −612 mV toward more noble side compared to −697 mV and −854 mV for heat-treated samples at 900 °C and 1200 °C respectively. Previous studies have proved that preferential attack on retained austenite phase in dual-phase steels, this was due to lower C content in retained austenite than in martensite phase^29,30^. Meaning that retained austenite phase will act as anode, while martensite phase will act as cathode in these dual-phase high-carbon steel samples. As austenitisation temperature increased, grain size of retained austenite and martensite will expand as well as increased amount of precipitation, thus, grain boundary surface contact will shrink. Another factor is due to presence of secondary phases on heat-treated steels and created a non-uniform effect on steel surface exposed to corrosion attack, i.e. pitting, grain boundary corrosion^31,32^.

**Table 2:** Tafel extrapolation parameters for different heat-treated high-carbon steel samples.

Sample-ID	E_corr_ (mV vs. SCE)	i_corr_ (mA/cm^2^)
base-material	−612 ± 6	0.4382 ± 0.0015
sample-900 °C	−697 ± 5	0.4749 ± 0.0019
sample-1200 °C	−854 ± 5	0.4813 ± 0.0009

On sample-1200 °C, the deposition of carbides on the grain boundaries leads to intergranular corrosion initiation. The carbides that precipitated near the grain boundaries causes micro strains and led to depletion of alloying elements near the boundaries making it susceptible to intergranular corrosion (IGC) attack^33^. MnS precipitations formed on surface of steel will initiate localised corrosion of steel matrix. Corrosion degradation was the quickest at inclusion or matrix interface which led to corrosion development surrounding inclusions^34^. This MnS existed on both steels after heat-treated high-carbon steels at temperature of 900 °C and 1200 °C. As results, occurrence of less numbers of MnS and more dominant MnC precipitations on sample-1200 °C had accelerated corrosion intensity and propagated intergranular attack (IGA) than on sample-900 °C and base-material.

### Nanoindentation hardness test

**Figure 11:**
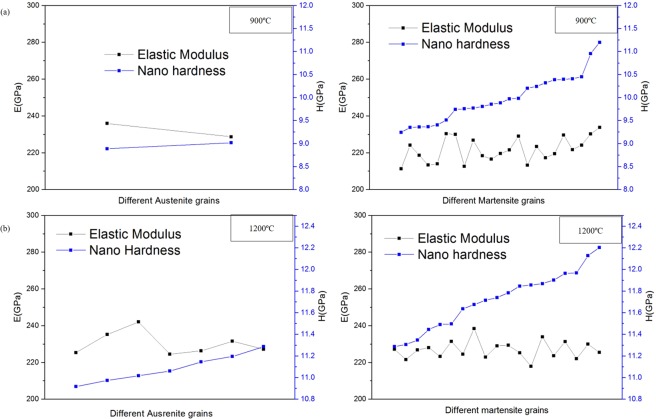
Comparison of elastic modulus (E) and nano hardness (H) values of different austenite and martensite grains on heated high-carbon steel samples at temperature of (**a**) 900 °C and (**b**) 1200 °C.

Hardness test was conducted to investigate the value difference of austenite and martensite phases after heat treatment process of high-carbon steel against base-material. Comparison of elastic modulus (E) and nano hardness (H) was presented in Fig. 11(a,b). Additionally, the summary of the average E and H values of each phase (austenite and martensite) at different temperature as shown in Tables 3 and 4. It can be observed that the variation in the temperature did not change the overall average hardness in different phases, i. e. martensite and austenite. However, the martensite phase showed considerable higher hardness compared to the austenite phase in all condition upon nano indentation because of the dislocation in martensite upon quenching. Martensitic structure formed from cooling from austenitic temperatures rapidly by pulling the heat out using a liquid quenchant before pearlite can form. Formation of martensite involved a transformation from a body-cantered cubic structure to body-centred tetragonal structure. Thus, a highly stressed structure formed due to the volume expansion. Therefore, martensite has a higher hardness than austenite for the exact same chemistry. The hardness of martensite increases with the increasing dislocation density. In contrast, austenite does not have any dislocation in the microstructure after quenching. In case of elastic modulus, it is an intrinsic property of the material which does not vary much from phase to phase. Before heat treatment, the hardness value of base-material high-carbon steel was measured to be 7.81 GPa in nanoindentation hardness testing. The average hardness at different martensite phase points revolved approximately 11.7 GPa, while retained austenite points around 11.1 GPa at sample-1200 °C, Thus, overall hardness of sample-1200 °C as measured from nanoindentation was around 11.4 GPa, which was significantly increased in comparison to base-material high-carbon steel sample. While, at the same time for sample-900 °C, average hardness value of martensite grains was found to be around 9.92 GPa and that of the retained austenite about 8.94 GPa, composing its average hardness of this steel sample to be 9.43 GPa. As samples were kept at austenitising temperature for 120 mins followed by rapid quenching, most of retained austenite had transformed into martensite, reducing the volume fraction of retained austenite to a mere few percentages. Increased hardness value of both heat-treated steel samples can be attributed to presence of spherical-like MnS inclusions, as it led to increase in hardness and nominal yield strength properties^35,36^. The original increased of hardness value on sample-1200 °C not only corresponded to austenisation temperature process. Additionally, the possible explanation to such substantial increase can be correlated to nano-sized carbides that must have formed in the matrix owing to sufficient energy provided by high temperature at 1200  °C. Thus, the primary micrometre-sized carbides and the secondary nanometre-sized carbides had significantly hardened the steel. These MnC precipitations had diffused and clustered on the grain boundaries, disrupting the motion of dislocations through steel, thereby escalating hardness value higher on sample-1200 °C than sample-900 °C.

**Table 3:** Average nano hardness (H) of the martensite and austenite phases at different temperature.

Average nano Hardness (GPa)/Temperature (°C)	900 °C	1200 °C
Martensite	255	256
Austenite	223	222

**Table 4:** Average elastic modulus (E) of the martensite and austenite phases at different temperature.

Average Elastic modulus (GPa)/Temperature (°C)	900 °C	1200 °C
Martensite	227	226
Austenite	228	228

## Conclusions

We have observed selective-precipitation phenomena on dual-phase low-alloy high-carbon steel *in-situ* at different austenitisation temperature through ultra-high temperature confocal scanning laser microscopy. From this acquired quantitative data, we have calculated the amount, average area of precipitations and ASTM grain size numbers at different temperature of 900 °C and 1200 °C, as a function of time. As the heating temperature increased, the value of ASTM grain size number decreased, and average grain size has increased. Micro-analysis of precipitations using SEM/EDS and EPMA confirmed the formation of MnS and MnC inclusions on both steel samples – with low concentration of grain boundary carbides, Fe_3_C, on sample-1200 °C. We measured the volume fraction of each phase using XRD analysis. From its crystallinity pattern, it was found that sample-900 °C possessed higher volume percentage of MnS than on sample-1200 °C. This different concentration of precipitant was depended on formation potential of each precipitant which is the function of isothermal holding time and quenching rate. From the electrochemical corrosion test average values indicated increase in corrosion rate as the heat treatment temperature increased from 900 °C to 1200 °C against the base-material. Nonetheless, nanoindentation hardness showed significant improvement as the temperature increased due to the strong presence of precipitations. Further studies at the micro size level is required to observe the minor changes as the effect of different inclusions on the steel samples. Although presence of these secondary phase precipitations had downgraded effect on corrosion resistance, as it will trigger early IGA, pit growth and grain boundary corrosion, but it will harden the steels, thus enhancing its hardness properties. Selective-precipitation mechanism is a simple and easy approach to use the production parameters and existing potential for precipitation of different phases in high-carbon steel to tailor the properties of this low-cost grade of steel for advanced applications.

## Data Availability

The data that support the findings of this study available from the corresponding author upon reasonable request.
